# Diabetic Retinopathy in Native and Nonnative Canadians

**DOI:** 10.1155/2007/76271

**Published:** 2008-01-16

**Authors:** Stuart A. Ross, Anne McKenna, Sheila Mozejko, Gordon H. Fick

**Affiliations:** ^1^Faculty of medicine, University of Calgary, # 238-4411 16th Ave NW, Calgary, AB, Canada T3B 0M3; ^2^Grant MacEwan College, 5-225K City Centre Campus, Edmonton, AB, Canada T5J 4S2; ^3^Department of Community Health, University of Calgary, 29th St Northwest, Calgary, AB, Canada T2N 1N4

## Abstract

High prevalence rates of type 2 diabetes are being observed in native Canadian communities. It is believed that native populations have a higher prevalence rate of vascular complications than nonnatives. The Southern Alberta Study of Diabetic Retinopathy (DR) examined the prevalence and incidence of DR and associated metabolic abnormalities in native and nonnative subjects. Prevalence rates of DR in type 2 diabetic native and nonnative subjects were identical, with a prevalence rate of 40%. Native subjects with retinopathy, however, tended to have more advanced changes of retinopathy compared to the nonnative subjects. Key factors such as A1c, blood pressure, duration of diabetes, and lipid values were not significantly different between the two cohorts. These data indicate that ethnicity does play a role in the development and severity of DR but potential risk factors that may affect the development of retinopathy are not significantly different between native and nonnative groups.

## 1. INTRODUCTION

Diabetic retinopathy remains an important cause of visual loss in persons with diabetes. As the prevalence rates of diabetes increase in both the native and nonnative populations, there is increasing concern of the potential increased numbers of patients at risk for diabetic retinopathy. Some potential risk factors for retinopathy include: A1c, BP (systolic and diastolic), and duration of diabetes [1–5]. Lipid abnormalities may also play part in the development of retinal changes [6].

It is often assumed that native populations have different prevalence rates of specific vascular complications and of the risk factors that may lead to the development of vascular complications. This paper reviews the prevalence rates of diabetic retinopathy in native and nonnative communities and directly compares the prevalence rates of potential risk factors for the development of diabetic retinopathy in these communities.

## 2. METHODOLOGY

The Southern Alberta Study of Diabetic Retinopathy (SASDR) was designed to provide assessment of the prevalence and incidence of diabetic retinopathy and other diabetes vascular complications and to assess potential risk factors for the development of retinopathy in a population of subjects with diabetes. Native and nonnative communities were assessed as part of the study. The study was carried out in southern Alberta and included both urban and rural areas and native reserves. At a minimum of three years following the completion of the initial prevalence study, all subjects were invited to take part in the incidence study, which repeated all parameters of the prevalence programme. This paper will review only the prevalence data.

All subjects were identified as either urban (Calgary) or rural, by the postal codes associated to their place of primary residence. Within the borders of the study area, there are six native reserves belonging to Treaty Seven, including the two largest native reserves in Canada, the Blood and Blackfoot reserves. The project was approved by (The Conjoint Health Research Ethics Board, University of Calgary, Canada) and by the medical committees of each of the native reserves. All southern Alberta reserves were represented in the study and are shown in Table 1.

### 2.1. Recruitment

**Insulin-using subjects**

Registration centers were established across the study area of southern Alberta. These included all pharmacies, physicians’ offices, nursing homes, auxiliary hospitals, native reserves and prisons. Each recruitment site had information and registration packages and staff was individually trained to administer the package to all subjects who used insulin.

**Noninsulin-using subjects**

The same techniques of registration could not be used for the noninsulin-using subjects as many may not use drugs or have the need to go into a pharmacy. Recruitment of the noninsulin-using subjects began after the identification of the insulin-using subjects. The family physician of each insulin-using subject registered in the study was provided with the number of insulin users registered from their practice and requested to invite the next and subsequent patients with noninsulin-using diabetes within the practice, to register for the study. Each recruitment site that had recruited insulin-using subjects was similarly asked to recruit an equal number of noninsulin-using subjects.

**Nonparticipants**

At each of the recruitment sites, if the diabetic subject refused to complete the registration card, the gender, approximate age of the subject, and the specific reason for the refusal was documented.

### 2.2. Assessment

Assessment of the individual subjects was undertaken in multiple sites. For those who lived in Calgary, participants attended the central SASDR office at the University of Calgary. For those who could not attend in Calgary, remote sites were set up across southern Alberta. At regular intervals, a mobile van was utilized to carry the staff and retinal and blood sampling equipment to the sites. Subjects were asked to complete an extensive 20-page questionnaire which included a review of ethnic origin, family, and personal medical history with a special emphasis on the history of diabetes, data of diagnosis, drug treatments, and existing vascular complications. A basic examination was performed, including height and weight for calculation of BMI; systolic blood pressure (BP-S) and diastolic blood pressure (BP-D). Each subject was also asked to provide a timed-overnight urine sample and a venous blood sample. Type 2 insulin-using subjects were identified by a serum C-peptide > 0.05 nmol/L.

**Ophthalmology assessment**

Each subject had a complete ophthalmology assessment including visual acuity, examination for cataracts, seven-field stereofundus retinal photography, and a slit lamp examination. The photographic slides were coded and forwarded to (Wisconsin Eye Reading Center, Wis, USA) for the classification of the degree of diabetic retinopathy according to the Airlie House classification as adapted by R. Klein et al. [7] Table 2.

**Laboratory investigations**

A venous blood sample was obtained for the measurement of a nonfasting lipid profile; A1c; serum creatinine; glucose; and serum C-peptide (for assessment of insulin-dependency). The urine sample was assessed for the presence of microalbuminuria and for the presence of ketones. The microalbumin-excretion rate was measured from a timed-overnight urine sample.

**Statistical analysis**

To describe the association of ethnicity with retinopathy and potential risk factors, chi-squared tests were used to compare rates and *t*-tests were used to compare means. All testing was done at the 0.05 level of significance.

## 3. RESULTS

**Retinopathy versus no retinopathy for native versus nonnative subjects**

There were a total of 2247 patients with Type 2 diabetes, 232 were native and 2015 were nonnative. Table 3 describes the breakdown of patients by ethnicity and retinopathy as follows.

The results of a Chi-Squared test for the equality of the retinopathy rates, (*χ* ^2^ = 0.0005 with 1 degree of freedom, *P* = .98) indicate that ethnicity appears to play no role on the incidence of retinopathy for patients with Type 2 diabetes.

The question of whether ethnicity is related to the degree of retinopathy is described in Table 4 as follows.

The Chi-Square test for independence (*χ* ^2^ = 13.52 with 2 degrees of freedom, *P* = .0002) indicates that there is a strong relationship between ethnicity and the degree of retinopathy for patients with Type 2 diabetes. If the patient is native there is a good chance that his/her retinopathy will be more severe than if the patient was nonnative.

**Risk factors for retinopathy**

Table 5 provides a breakdown of these factors relative to native/nonnative classifications as follows.

The native subjects had poorer glucose control with significantly higher levels of A1c compared to the nonnative subjects. There were small but significant differences between native and nonnative subjects in measurements of diastolic blood pressure. Native subjects showed significantly better lipid profiles compared to the nonnative subjects. There were no significant differences in systolic blood pressure measurements or duration of diabetes between the two groups.

Tables 6–9 investigate the risk factors with degree of retinopathy. Each table is broken down by the risk factor and native/nonnative categories. Ret 1 is no retinopathy; Ret 2 is background retinopathy; Ret 3 is preproliferative retinopathy; and Ret 4 is proliferative retinopathy.

Native subjects had significantly higher A1c values with minor differences in the other parameters Table 6.

Native subjects had significantly higher BP-D; HDL-C values; and shorter duration of diabetes Table 7.

There were no clinically significant differences between the native and nonnative groups apart from significantly higher HDL-C values in native subjects Table 8.

While the mean values in the native group for A1c, blood pressure readings, lipids, and duration are higher than the nonnative groups, there were no significant differences between the two subject groups Table 9.

The most frequent differences in risk factors between native and nonnatives are at the “no retinopathy” stage and the “background” stage. Once a patient has preproliferative or proliferative disease, there is very little or no difference in risk factors regardless of ethnicity.

## 4. DISCUSSION

The prevalence of diabetes is increasing worldwide. Associated with this increase is an increased risk of development of micro- and macrovascular complications including diabetic retinopathy. Aboriginal or native communities may be at increased risk of developing prediabetes or type 2 diabetes. There may well be an increased risk of microvascular complications amongst these communities [8–13].

Native communities in north America have previously been identified as having an increased risk of type 2 diabetes [14]. While the risk of developing type 2 diabetes may vary between various native-Canadian nations, many are exhibiting an increased prevalence rate of type 2 diabetes [13, 15–21]. Detailed studies of (Ojibwa-Cree native populations in Sandy Lake, Ontario, Canada) have revealed increased prevalence rates of impaired glucose tolerance as well as type 2 diabetes [8, 10, 22]. Similar abnormalities have been noted in (James Bay Cree in northern Québec, Canada) [19].

Native Canadians have also been identified as having increased morbidity and mortality associated with diabetes with a greater impact on quality of life and a strong association between diabetes, hypertension, heart disease, and sight impairment [9]. Renal disease is also more prominent with an increased prevalence rate of diabetic end-stage renal disease evident in native Canadians [23–26].

There are only a limited number of studies related to diabetic retinopathy in native populations. In Canada particularly, many native populations are in remote areas and extensive studies have proven to be difficult. Increased vascular complications, including diabetic retinopathy, have been noted in the James Bay Cree and other native populations [24, 25, 27, 28]. Haffner et al. noticed an increase prevalence rate of diabetic retinopathy in Mexican-American diabetic subjects compared to Caucasian diabetic subjects reviewed in (Wisconsin Epidemiologic Study of Diabetes, Wis, USA) suggesting an ethnicity effect on the risk of developing diabetic retinopathy [29]. Studies of the Pima Indians have shown a similar increase in prevalence rates [30]. An urban study, however, carried out in the native population in Vancouver, showed that visual disability was nine times greater than in the general Canadian population but diabetic retinopathy was not a major cause of that visual loss [31].

Various risk factors have been identified as leading to an increased incidence of diabetic retinopathy. These include glycemic control [4, 5, 32–35], elevated blood pressure [3, 33, 36–38], abnormal lipids [39, 40], and duration of diabetes [41]. It is thought that native Canadians may exhibit higher prevalence rates of these risk factors, compared to nonnative individuals. Detailed studies of native-north Americans have revealed high prevalence rates of these risk factors. A review of Sandy Lake community demonstrated high prevalence rates of vascular complications and associated risk factors [8, 11]. Studies of other native Canadian communities have revealed similar results [18, 42, 43].

The prevalence data from the Southern Alberta Study of Diabetic Retinopathy (SASDR) provides a unique opportunity to review prevalence rates of diabetic retinopathy, severity and the potential associated risk factors for the development of retinopathy directly between insulin and noninsulin using type 2 diabetic native and nonnative subjects.

As can be seen from the data presented, there were no differences in prevalence rates of retinopathy between the native and nonnative groups. Native subjects with retinopathy, however, tended to have more advanced changes of retinopathy compared to the nonnative subjects.

When assessing the role of potential risk factors for the development or progression of diabetic retinopathy, again, there were only few and minor differences between native and nonnative subjects. Both native and nonnative subjects with more severe presentation of diabetic retinopathy tended to have higher levels of A1c compared to those with less severe forms of retinopathy. Significantly higher levels of A1c were observed only in native subjects who did not exhibit retinopathy. A1c was not statistically significant for patients with some form of retinopathy. Native subjects tended to have lower total-cholesterol and higher HDL-C values compared to nonnative groups although these differences were small. Duration of diabetes, which is recognized as a major risk factor for the development and severity of diabetic retinopathy, was not significantly different between native and nonnative subjects.

Of interest are the differences in prevalence and severity of diabetic retinopathy in the southern Alberta native subjects compared to those from the detailed Sandy Lake study [11]. The prevalence rates of retinopathy were far higher in the southern Alberta cohort and similar to those identified in the Wisconsin Eye Studies [44].

These data indicate that ethnicity does play a significant role in the development and severity of diabetic retinopathy but potential risk factors that may accelerate the development of retinopathy are not significantly different between native and nonnative groups.

## Figures and Tables

**Table 1:** Southern Alberta native reserves-treaty seven.

Reserve	Tribe
Blood	Blackfoot
Piikani	Blackfoot
Blackfoot	Blackfoot
Morley	Stoney
Eden Valley	Stoney
TsuuTina	Sarcee

**Table 2:** Retinal grade classification.

Grade	Description
10–14	no retinopathy
15–21	microaneurysms or soft exudates or retinal hemorrhages
31	micro aneurysms with any of: venous loops; soft exudates; intraretinal microvascular abnormalities (IRMA); venous beading; retinal hemorrhages
41	microaneurysms with any of the following: hemorrhages and 4-5 fields; IRMA 1–3 fields
51	microaneurysms with one or more of the following: hemorrhages and microaneurysms in 4-5 fields; IRMA in 4 or more fields; venous beading in 2 or more fields
60	fibrous proliferations
61	scars of photocoagulation but no evidence of 60 or 65
65	proliferative retinopathy

**Table 3:** Prevalence of diabetic retinopathy.

	Native	Nonnative	Total
No retinopathy	137 (60%)	1208 (60%)	1345 (60%)
retinopathy	91 (40%)	805 (40%)	895 (40%)

Total	228 (100%)	2013 (100%)	2241(100%)

**Table 4:** Degree of retinopathy.

	Native	Nonnative	Total
Background	45 (49%)	551 (68%)	596 (67%)
Preproliferative	33 (36%)	174 (22%)	207 (23%)
Proliferative	13 (14%)	80 (10%)	93 (10%)

Total	91 (100%)	805 (100%)	896 (100%)

**Table 5:** Risk factors for diabetic retinopathy.

Factor	Observations		Average		Standard deviation		*P*
	Native	Nonnative	Native	Nonnative	Native	Nonnative	
A1c	224	2010	7.52	7.15	2.0	1.78	.008
BP-S mm/Hg	228	2010	129	130	21.59	19.07	.3611
BP-D mm/Hg	228	2010	78	75	10.27	10.11	.0025
Total-Cholesterol mmol/L	225	2011	5.4311	5.5454	1.12	1.32	.0274
HDL-C mmol/L	225	2007	1.1748	1.056	0.37	0.32	<.0005
Duration years	221	1981	8.26	9.08	7.13	8.42	.164

**Table 6:** No retinopathy and associated risk factors.

Ret1	Observations		Average		Standard deviation		*P*
	Native	Nonnative	Native	Nonnative	Native	Nonnative	
A1c	134	1205	7.20	6.85	1.89	1.67	.0455
BP-S mm/Hg	137	1205	125	127	18.3	17.8	.1262
BP-D mm/Hg	137	1205	77	75	9.2	10	.0544
Total-Cholesterol mmol/L	135	1206	5.323	5.533	1.02	1.32	.0733
HDL-C mmol/L	135	1202	1.182	1.055	0.385	0.312	.003
Duration years	133	1190	5.962	7.086	5.95	7.65	.1015

**Table 7:** Background retinopathy and associated risk factors.

Ret2	Observations		Average		Standard deviation		*P*
	Native	Nonnative	Native	Nonnative	Native	Nonnative	
A1c	45	551	7.74	7.37	1.77	1.67	.1596
BP-S mm/Hg	45	551	131	133	20.5	19.9	.662
BP-D mm/Hg	45	551	80	76	11.3	9.9	.01
Total-Cholesterol mmol/L	45	551	5.282	5.513	1.01	1.26	.2291
HDL-C mmol/L	45	551	1.178	1.076	0.391	0.335	.0524
Duration years	40	535	8.6	11.215	5.56	8.51	.0564

**Table 8:** Preproliferative retinopathy and associated risk factors.

Ret3	Observations		Average		Standard deviation		*P*
	Native	Nonnative	Native	Nonnative	Native	Nonnative	
A1c	33	174	8.03	8.17	2.0	1.89	.7006
BP-S mm/Hg	33	174	77	77	11.4	10.1	.965
BP-D mm/Hg	33	174	133	137	26.3	20.6	.4216
Total-Cholesterol mmol/L	33	174	5.376	5.731	1.6	1.53	.2254
HDL-C mmol/L	33	174	1.166	1.022	0.302	0.297	.0119
Duration years	33	174	13.939	13.362	6.99	8.24	.7064

**Table 9:** Proliferative retinopathy and associated risk factors.

Ret4	Observations		Average		Standard deviation		*P*
	Native	Nonnative	Native	Nonnative	Native	Nonnative	
A1c	12	80	8.76	7.88	2.55	1.55	.2596
BP-S mm/Hg	13	80	147	136	32.4	20.7	.2577
BP-D mm/Hg	13	80	80	76	13.8	12.7	.2593
Total-Cholesterol mmol/L	12	80	5.7108	5.546	1.17	1.31	.6828
HDL-C mmol/L	12	80	1.113	1.01	0.338	0.249	.208
Duration years	13	80	16.769	15.4	9.2	9.25	.6217
